# Robust Model-Free Identification of the Causal Networks Underlying Complex Nonlinear Systems

**DOI:** 10.3390/e26121063

**Published:** 2024-12-06

**Authors:** Guanxue Yang, Shimin Lei, Guanxiao Yang

**Affiliations:** 1 School of Electrical and Information Engineering, Jiangsu University, Zhenjiang 212013, China; 2222207067@stmail.ujs.edu.cn; 2 College of Automation, Jiangsu University of Science and Technology, Zhenjiang 212100, China; dxxy@just.edu.cn

**Keywords:** model-free, data-driven, causal inference, Granger causality, nonlinear dynamics

## Abstract

Inferring causal networks from noisy observations is of vital importance in various fields. Due to the complexity of system modeling, the way in which universal and feasible inference algorithms are studied is a key challenge for network reconstruction. In this study, without any assumptions, we develop a novel model-free framework to uncover only the direct relationships in networked systems from observations of their nonlinear dynamics. Our proposed methods are termed multiple-order Polynomial Conditional Granger Causality (PCGC) and sparse PCGC (SPCGC). PCGC mainly adopts polynomial functions to approximate the whole system model, which can be used to judge the interactions among nodes through subsequent nonlinear Granger causality analysis. For SPCGC, Lasso optimization is first used for dimension reduction, and then PCGC is executed to obtain the final network. Specifically, the conditional variables are fused in this general, model-free framework regardless of their formulations in the system model, which could effectively reconcile the inference of direct interactions with an indirect influence. Based on many classical dynamical systems, the performances of PCGC and SPCGC are analyzed and verified. Generally, the proposed framework could be quite promising for the provision of certain guidance for data-driven modeling with an unknown model.

## 1. Introduction

Grasping the structure and dynamics of a networked system is crucial for understanding the functionality and influence mechanisms of these systems, which could lay the foundation for better controlling complex networked systems. To date, advances in data science and machine learning have promoted the prosperity and development of network science. As is well known, networks are represented not only by their structures, but also by the dynamical information taking place on the structures. Network reconstruction, as an important aspect of research on complex networks, has attracted the attention of many researchers from various fields, including biological, electrical, physical, financial, and ecological sciences, the study of social networks, and so on [1,2,3,4,5]. Multidisciplinary studies have led to the emergence of technological means based on the understanding and analysis of big data. However, the transient evolution of real complex systems is often accompanied by high complexity and nonlinearity. There is, in actuality, a lack of unified physical models for dynamical processes from complex data, where the dynamical laws underlying the real systems are usually elusive. Hence, in view of the large amount of data and complex laws, data-driven modeling and causal discoveries from system dynamics are expected to play an important part in the era of big data.

Over the last decade, a great deal of methods have been developed to infer networks from observational datasets, such as ordinary differential equations, nonlinear dynamic modeling methods, similarity-based methods, Granger causality, and so on. Many of these methods suppose that a model of the system dynamics is known in advance. Based on predefined or known mathematical models, the identification of undetermined parameters or the inference of network topology is relatively easy [5,6,7,8]. The methods based on these models sometimes do not work well, as most related modeling assumptions and conditions may not be satisfied and may be unavailable in real experimental studies. In linear dynamic modeling, many methods for network reconstruction only adopt a linear model to fit the data. These methods only consider the dynamical evolution of complex systems under a linear ordinary differential equation [9,10]. If the dynamic trajectories of a system vary from transients toward steady states, some of the related methods could also involve a linear regression method based on the datasets of steady states [11]. In actuality, these linear modeling methods cannot satisfy the demands and characteristics of complex nonlinear networked systems. As such, an adaptive differentiation scheme and pre-filtration step were introduced to linear ordinary differential equations, in order to tackle the challenge of inferring large-scale complex nonlinear networks, and this method was able to outperform some current state-of-the-art methods [12]. However, there is still a great deal of work to be carried out on network inference for complex nonlinear systems.

For unknown complex dynamical systems, many nonlinear dynamic modeling methods have been presented, and the nonlinear forms are different for different applications. Specifically, some works—which intend to build spaces of all possible basis functions for the subsequent model selection with sparse-promoting methods, if such nonlinear system dynamics can be sparsely represented by the proposed basis functions—have developed a novel framework to identify a dynamical system governed by nonlinear equations [13,14,15]. Although these methods could resort to using some basic knowledge and information about the physical laws of specific systems, it is worth mentioning that the reasonable choice of the function basis is not always explicit. Another approach determines the topology of networks based on the assumption that the network dynamics are a combination of self-dynamics and coupling dynamics, the crucial idea of which is to approximate a node’s self-dynamics and coupling dynamics with complete orthogonal bases (e.g., the Legendre or Chebyshev polynomials or the Fourier basis) [16]. Furthermore, a novel model-free inference method was posed as a multivariate regression problem by systematically decomposing each unit’s dynamics into pairwise, three-point, and higher-order interactions with other units in the network, as well as by introducing an explicit dependency matrix in combination with a block-orthogonal regression algorithm [17]. However, it is inevitable that this method would also require the network dynamics to be additive among multiple-order interactions and, sometimes, the system’s dynamical model will not be able to decompose combinations of multiple-order interacting items. Therefore, these types of assumptions are restricted to some real fields of science and engineering.

In addition, similarity-based indicators are the basis of some popular methods in statistical science, the basic theories and technologies of which are correlation coefficients, mutual information, transfer entropy, and so on. They are usually used to extrapolate the causality and correlation among datasets. For pairwise relationships, there exist correlation coefficients and mutual information for linear and nonlinear cases, respectively. However, these methods result in indirect links when the number of nodes increases. As such, partial mutual information and partial correlation coefficients, which are designed to silence indirect relationships through the influence of common external inputs induced by other nodes in the whole network, have been proposed for this purpose [18,19,20]. In addition, these correlation coefficient and mutual information methods are only designed based on the consideration of the correlation, which cannot be used for causal inference. Hence, transfer entropy based on information theory was developed to uncover causality using time series data. Transfer entropy was first proposed to find the nonlinear causal interactions between bivariate time series [21]. Then, conditional transfer entropy [22,23] was put forward to eliminate the indirect influence of other variables in the multivariate case. For information theory methods, when the dimension of the variable increases, the calculation of the probability density function becomes difficult and complicated. Moreover, when used for the general judgment of statistical dependencies, the ability of these similarity-based methods should be further improved for specialized fields.

As an equivalent companion of transfer entropy for Gaussian variables [24], Granger causality is another popular and powerful causality detection method that is usually designed to find the temporal interactions among time–course variables in complex multivariate systems [25,26,27,28]. As a heuristic statistical prediction concept based on the vector autoregressive (VAR) model, Granger causality was originally presented in econometrics with the aim of determining whether or not the past observations of a process help to predict the future values of another process [29]. Accordingly, conditional Granger causality was introduced to distinguish direct interactions from indirect ones [30]. Then, formulations of Granger causality in the frequency domain were proposed, such as directed transfer functions, partially directed coherence, and so on [31,32,33]. In order to deal with nonlinear relationships, some nonlinear Granger causality methods have also been proposed based on some kernel functions and copula theory, such as kernel Granger causality [34,35,36] and copula Granger causality [37].

In order to make up for the deficiency of current Granger causality methods, recent works on Granger causality are still at the forefront of scientific research on network inference [38,39,40]. Additionally, in order to deal with the problem of small sample sizes for large-scale networks, many sparse-promoting methods can be applied to traditional Granger causality methods, such as truncated Lasso Granger causality [41], grouped Lasso graphical Granger [42], and an improved backward conditional Granger causality (mBTSCGC) [43]. Direct comparisons of many penalized regression approaches have been extensively conducted to provide guidelines for further applications [44]. However, these sparse Granger causality methods are mainly proposed based on linear VAR models. For the consideration of nonlinear modeling, group Lasso nonlinear conditional Granger causality (GLasso-NCGC) was proposed for nonlinear causal inference [45], the nonlinear formulation of which is only for certain types of systems. As the nonlinear variant of mBTSCGC, rpCGCI was further proposed, where the mBTS scheme was applied to the polynomial VAR model for Granger causality inference [46]. However, rpCGCI only focuses on model formulation up to second-order polynomial products, and the second-order polynomial approximation is not sufficient for highly complex nonlinear systems. Furthermore, LASSO regression was used in a VAR framework and state-space model to compute the information dynamics [47]. Then, artificial neural networks (ANNs) were combined with state-space models for Granger causality analysis [48].

In this study, we put forward a new and unified framework for identifying causal networks and predicting nonlinear dynamics from noisy observational datasets in complex nonlinear systems. Rather than identifying parameters of complex systems governed by predefined and known models or taking some polynomial, rational, or other basis functions as prior information for the subsequent model selection, we first propose Granger causality based on the general nonlinear dynamical framework, which is termed Polynomial Conditonal Granger Causality (PCGC) and Sparse Polynomial Conditional Granger Causality (SPCGC); these not only consider the nonlinearity and causality of mutual interactions, but also reconcile the inference of direct interactions with the influence of indirect links. In particular, in our PCGC model, the polynomial approximation can be extended to multiple-order products for the demands of highly complex nonlinear systems, which are not limited to the second order. The network dynamics in our nonlinear framework are not simply additive between the self-dynamics and the coupling dynamics (pairwise, three-point, and other higher-order interactions among nodes in the network). We investigate our approach in many different types of complex nonlinear systems and verify the performance of several standard classical dynamical models, the system trajectories of which contain dynamical behaviors from transients towards steady states and from transients to periodic dynamics, in addition to non-periodic dynamical trajectories and chaotic dynamical trajectories. All of the simulation results demonstrate the effectiveness and robustness of the proposed method when there is a specific polynomial order and a proper size of observations.

## 2. Materials and Methods

### 2.1. Polynomial Conditional Granger Causality Model

The dynamical behavior of complex networked systems is governed by the interactions among a population of units (nodes). For each state variable in a complex system, the dynamical response or evolution is determined by the driving variables on the right side of the equation.

In general, nonlinear dynamical systems can be described using the following general differential equations:

(1) $$\left\{\begin{array}{c} {\dot{x}}_{1}=f_{1}\left(x_{1},x_{2},\ldots ,x_{n}\right)\\ {\dot{x}}_{2}=f_{2}\left(x_{1},x_{2},\ldots ,x_{n}\right)\\ \vdots \\ {\dot{x}}_{n}=f_{n}\left(x_{1},x_{2},\ldots ,x_{n}\right) \end{array}\right.,$$

where $x_{i}$ is the state variable and i=1,2,…,n; *n* is the dimension of the system; ${\dot{x}}_{i}$ describes the time derivative of $x_{i}$; and the nonlinear function $f_{i}\left(•\right)$ represents the dynamical law of the system.

In detail, for each target variable $x_{i}$, Equation (2) can be obtained as follows:

(2) $${\dot{x}}_{i}=f_{i}\left(x_{1},x_{2},\ldots ,x_{n}\right).$$

Obviously, this is a unified manner of establishing a nonlinear framework for all complex networked systems. In order to investigate the causal relationship between the set of driving variables $\left\{x_{1},x_{2},\ldots ,x_{n}\right\}$ and the target variable $x_{i}$, the exact identification of the nonlinear function $f_{i}\left(•\right)$ is of crucial importance. Here, we use the *r*-th-order polynomial function to fit the nonlinear function $f_{i}\left(•\right)$. Generally, the nonlinear function $f_{i}\left(x_{1},x_{2},\ldots ,x_{n}\right)$ can consist of polynomials from the driving variable set $\left\{x_{1},x_{2},\ldots ,x_{n}\right\}$. Then, $f_{i}\left(x_{1},x_{2},\ldots ,x_{n}\right)$ can be denoted as shown in Equation (3).

(3) $$\begin{array}{cc} f_{i}\left(x_{1},x_{2},\ldots ,x_{n}\right) & =\Phi a_{i}\\ \; & =\sum_{k_{1},k_{2},\ldots ,k_{n}}a_{k_{1},k_{2},\ldots ,k_{n}}x_{1}^{k_{1}}x_{2}^{k_{2}}\cdots x_{n}^{k_{n}}, \end{array}$$

where Φ is a vector of polynomial basis; $a_{i}$ is an unknown parameter; $\{k_{1},k_{2},\ldots ,k_{n}\}\subset N$; and $k_{1}+k_{2}+\cdots +k_{n}\leq r$. *r* is the order of the polynomial. In detail, the candidate function library Φ can be expressed as follows:

(4) $$\begin{array}{c} \Phi =\left[\begin{array}{cccccc} 1 & x^{p_{1}} & x^{p_{2}} & x^{p_{3}} & \cdots & x^{p_{r}} \end{array}\right], \end{array}$$

where $x^{p_{r}}$ can be taken to obtain the *r*-th-order polynomial, which includes the set of *r*-th-order monomials relating to $\left\{x_{1},x_{2},\ldots ,x_{n}\right\}$. Here, $x^{p_{r}}$ is defined as the *r*-th-order polynomial vector. For example, the first-order vector and the second-order vector can be described as follows:

$$\begin{array}{cc} x^{p_{1}} & =[\begin{array}{cccc} x_{1} & x_{2} & \cdots & x_{n} \end{array}],\\ x^{p_{2}} & =\left[\begin{array}{cccccccc} x_{1}^{2} & x_{1}x_{2} & \cdots & x_{1}x_{n} & x_{2}^{2} & x_{2}x_{3} & \cdots & x_{n}^{2} \end{array}\right]. \end{array}$$

Other higher-order polynomial vectors (e.g., $x^{p_{3}}$, $x^{p_{4}}$, etc.) can be expressed similarly. As shown in Equation (4), when the polynomial order *r* is given, Φ includes all related polynomial items whose order is less than or equal to *r*.

Next, based on the theory of Granger causality, our nonlinear causal index is proposed for a nonlinear dynamical system as follows. For Equation (2), the complete set of all variables in the system is denoted as $\Gamma =\{x_{1},x_{2},\ldots ,x_{n}\}$. For each target variable $x_{i}$, the unrestricted model can be described as shown in Equation (5).

(5) $${\dot{x}}_{i}=f_{i}\left(\Gamma \right)+\xi_{i}.$$

In order to judge which driving variable $x_{j}$ in Γ causally influences the target variable $x_{i}$, the restricted model can be written as shown in Equation (6).

(6) $${\dot{x}}_{i}=f_{i}\left(\Gamma \backslash x_{j}\right)+\xi_{j},$$

where $\Gamma \backslash x_{j}$ means removing the variable $x_{j}$ in Γ, i.e., $\Gamma \backslash x_{j}=\left\{x_{1},x_{2},\ldots ,x_{j-1},x_{j+1},\ldots ,x_{n}\right\}$. Accordingly, for the nonlinear function $f_{i}\left(\Gamma \backslash x_{j}\right)$, all relevant items of $x_{j}$ in $f_{i}\left(\Gamma \right)$ are removed. Then, the residual $\xi_{i}$ of the unrestricted model and the residual $\xi_{j}$ of the restricted model are calculated. In this case, it is worth noting that the inference of the causal relationship between $x_{j}$ and $x_{i}$ is conditioned on the set of other nodes in the network $\Gamma \backslash x_{j}$, which is designed to silence the indirect relationships through the influence of common external inputs induced by other nodes in the entire network. The conditional variables are not just explicitly added to the driving variable, but they are latently integrated into the nonlinear function, which has already involved the driving variable.

Finally, the nonlinear causal index (PCGC) is calculated as follows:

(7) $${\mathrm{PCGC}}_{x_{j}\to x_{i}}=\ln\frac{\sigma_{j}^{2}}{\sigma_{i}^{2}},$$

where $\sigma_{i}^{2}$ and $\sigma_{j}^{2}$ are the variances in error $\xi_{i}$ and $\xi_{j}$, respectively. Generally, if PCGC is greater than the given threshold value δ, this confirms that $x_{j}$ causes $x_{i}$, i.e., there exists a link $x_{j}\to x_{i}$.

In theory, an unrestricted model with more parameters will always be able to fit the data at least as well as a restricted model with fewer parameters. So, an unrestricted model with lower error will result in a better fit to the data than a restricted model. Meanwhile, in order to determine whether the unrestricted model gives a significantly better fit to the data, the F-statistic hypothesis is taken when obtaining the final network. The F-statistic value should be smaller than the given significance level $P_{val}$. The formulation of the F-statistic hypothesis is defined as

(8) $$F=\frac{\left({∥\xi_{i}∥}^{2}-{∥\xi_{j}∥}^{2}\right)⁄\left(n_{i}-n_{j}\right)\;}{{∥\xi_{i}∥}^{2}⁄\left(n_{i}-m\right)\;},$$

where ${∥\xi_{i}∥}^{2}$ and ${∥\xi_{j}∥}^{2}$ are the residual sums of squares in the unrestricted model and the restricted model, respectively. $n_{i}$ and $n_{j}$ are the total numbers of parameters in the unrestricted and restricted models, respectively. *M* is the number of observations.

In order to determine the interactions among variables in the system, all of the time histories of state variables $x_{i}$ (i=1,2,…,n) are collected, and the derivatives ${\dot{x}}_{i}$ can be measured directly or approximated using several methods, such as the Euler and Runge–Kutta methods.

Furthermore, suppose that the data are sampled several times, and *T* time points are obtained at each measurement. The state matrix X and derivative matrix $\dot{X}$ are collected as follows:

$$\begin{array}{c} \dot{X}=\left[\begin{array}{cccc} {\dot{x}}_{1,1} & {\dot{x}}_{2,1} & \cdots & {\dot{x}}_{n,1}\\ {\dot{x}}_{1,2} & {\dot{x}}_{2,2} & \cdots & {\dot{x}}_{n,2}\\ \vdots & \vdots & \ddots & \vdots \\ {\dot{x}}_{1,T} & {\dot{x}}_{2,T} & \cdots & {\dot{x}}_{n,T} \end{array}\right]\;and\;X=\left[\begin{array}{cccc} x_{1,1} & x_{2,1} & \cdots & x_{n,1}\\ x_{1,2} & x_{2,2} & \cdots & x_{n,2}\\ \vdots & \vdots & \ddots & \vdots \\ x_{1,T} & x_{2,T} & \cdots & x_{n,T} \end{array}\right], \end{array}$$

where $x_{i,t}$ denotes the data point of $x_{i}$ that is sampled at moment *t*, and ${\dot{x}}_{i,t}$ denotes the value of the derivative ${\dot{x}}_{i}$ that is measured or estimated at moment *t*.

Sometimes, in order to ensure a variety of datasets, multiple measurements are carried out under different conditions (random initial values, adding perturbations, removing nodes, etc.). Here, the number of multiple measurements is set to *m*. Then, the total number of observations is set to M=mT. Then, data matrices ($\dot{X}$ and X) with M×n are collected, i.e.,

$$\dot{X}=\left[\begin{array}{c} {\dot{X}}^{\left[1\right]}\\ {\dot{X}}^{\left[2\right]}\\ \vdots \\ {\dot{X}}^{\left[m\right]} \end{array}\right]\;and\;X=\left[\begin{array}{c} X^{\left[1\right]}\\ X^{\left[2\right]}\\ \vdots \\ X^{\left[m\right]} \end{array}\right],$$

where, at the *m*-th measurement, ${\dot{X}}^{\left[m\right]}$ and $X^{\left[m\right]}$ are denoted as

$$\begin{array}{c} {\dot{X}}^{\left[m\right]}=\left[\begin{array}{cccc} {\dot{x}}_{1,1}^{\left[m\right]} & {\dot{x}}_{2,1}^{\left[m\right]} & \cdots & {\dot{x}}_{n,1}^{\left[m\right]}\\ {\dot{x}}_{1,2}^{\left[m\right]} & {\dot{x}}_{2,2}^{\left[m\right]} & \cdots & {\dot{x}}_{n,2}^{\left[m\right]}\\ \vdots & \vdots & \ddots & \vdots \\ {\dot{x}}_{1,T}^{\left[m\right]} & {\dot{x}}_{2,T}^{\left[m\right]} & \cdots & {\dot{x}}_{n,T}^{\left[m\right]} \end{array}\right]\;and\;X^{\left[m\right]}=\left[\begin{array}{cccc} x_{1,1}^{\left[m\right]} & x_{2,1}^{\left[m\right]} & \cdots & x_{n,1}^{\left[m\right]}\\ x_{1,2}^{\left[m\right]} & x_{2,2}^{\left[m\right]} & \cdots & x_{n,2}^{\left[m\right]}\\ \vdots & \vdots & \ddots & \vdots \\ x_{1,T}^{\left[m\right]} & x_{2,T}^{\left[m\right]} & \cdots & x_{n,T}^{\left[m\right]} \end{array}\right]. \end{array}$$

Next, for the target variable $x_{i}$, the matrix formulation of Equation (2) is shown in Equation (9).

(9) $${\dot{X}}_{i}=f\left(X\right)=\Theta a_{i}+\Xi_{i},$$

where ${\dot{X}}_{i}={\left[\begin{array}{ccccccc} {\dot{x}}_{i,1}^{\left[1\right]} & \cdots & {\dot{x}}_{i,T}^{\left[1\right]} & \cdots & {\dot{x}}_{i,1}^{\left[m\right]} & \cdots & {\dot{x}}_{i,T}^{\left[m\right]} \end{array}\right]}^{T}$ is the *i*-th column of $\dot{X}$, which corresponds to the derivative data of variable *i*. Θ is a nonlinear data matrix under the polynomial basis Φ. $a_{i}$ is the coefficient to be solved. $\Xi_{i}$ is the vector of the model error.

**Figure 1:**
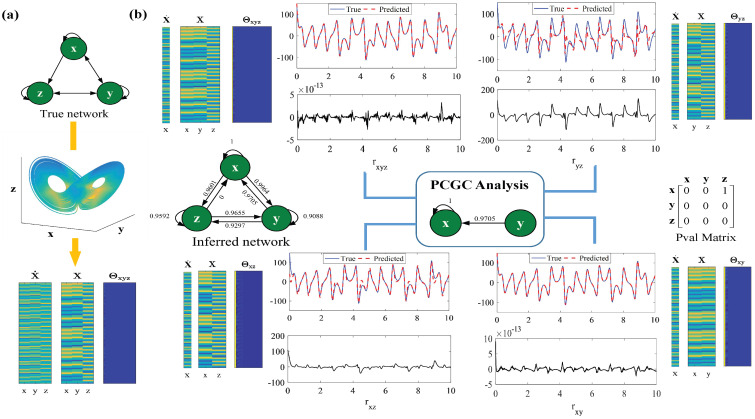
A flowchart of Polynomial Conditional Granger Causality based on the results of a simulation of a Lorenz oscillator system. (**a**) Datasets (X and $\dot{X}$) are generated from the Lorenz system equations. The noise strength σ is set to zero. Here, m=1 and T=2000; that is, data are collected from the interval $t\in \left[0,20\right]$ with the time step Δt=0.01. Θ is a nonlinear data matrix with the polynomial basis Φ. The unrestricted model of the Lorenz system is initially constructed in the complete space of polynomials in (x,y,z) up to the fifth order, and the corresponding nonlinear data matrix is $\Theta_{xyz}$. (**b**) For the target variable *x*, the derivative vector $\dot{x}$ is arranged to be the output variable of both the unrestricted model and the restricted model. Then, three restricted models are obtained to judge which driving variable causally influences the target variable. In detail, the restricted models of the system are constructed in the subspace of polynomials in (y,z), (x,z), and (x,y) up to the fifth order. The corresponding nonlinear data matrixes are $\Theta_{yz}$, $\Theta_{xz}$, and $\Theta_{xy}$, respectively. The dynamic trajectories of *x* in different cases are plotted, and the respective errors of prediction are analyzed. The Lorenz oscillator system is shown in Equation (A1) in Appendix A.

Taking a Lorenz oscillator system as an example, a flowchart of Polynomial Conditional Granger Causality based on the results of a simulation of this classical chaotic system is illustrated in Figure 1. Firstly, a benchmark network is obtained from the equation of the Lorenz oscillator system for comparison. In order to obtain the inferred network, the identification of the whole network is decomposed into the selection of neighbors for each target node. For example, to investigate the relationship of *x* with all of the variables in the system (a self-loop is also considered), unrestricted and restricted regression models are jointly constructed. In detail, to explore the impact of the driving variable *y* on the target variable *x*, an unrestricted model of the complete space of polynomials in (x,y,z) and a restricted model of the subspace of polynomials in (x,z) are constructed. The corresponding dynamic trajectories of *x* predicted by the unrestricted model in (x,y,z) and the restricted model in (x,z) are illustrated. From the errors of regression ($r_{xyz}$ and $r_{xz}$), we find that the accuracy of curve fitting decreases when the past information of *y* is excluded. Hence, the causal inference from *y* to *x* (y→x) is found. Similarly, the corresponding dynamic trajectories of *x* predicted by the restricted model in (y,z) and (x,y) are also plotted. The accuracy sharply decreases when the past information of *x* is removed. In this case, the self-loop of *x* is inferred. However, high accuracy is also obtained without the past information of *z*, which means that *z* is not the cause of *x*. So, a subnetwork relating to target node *x* is formed. In the same way, the entire network is obtained, and the Granger causal strength is marked on each link by calculating the nonlinear causal index. Meanwhile, the $P_{val}$ matrix is obtained based on the F-statistic test. The significance level $P_{val}$ is found to be $10^{-10}$. In Figure 1, we find that the inferred network perfectly matches the benchmark network, with high causal strength and high confidence.

### 2.2. Sparse Polynomial Conditional Granger Causality

**Figure 2:**
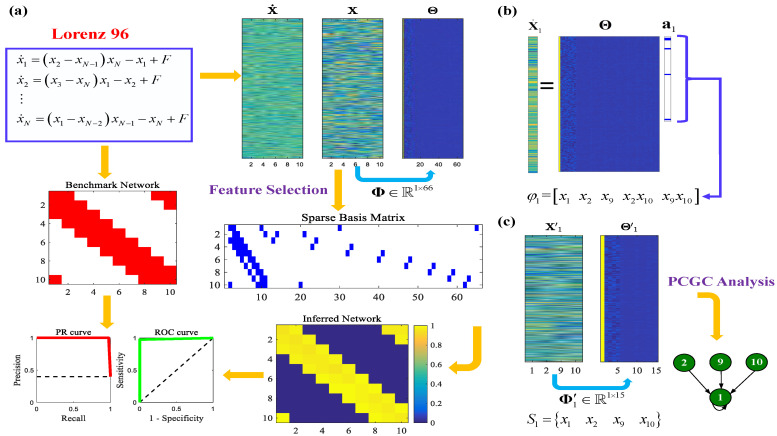
A flowchart of Sparse Polynomial Conditional Granger Causality based on the results of the simulation of a Lorenz 96 system with N=10. Here, m=300, T=3, r=2, λ=0.001, and $P_{val}=10^{-10}$. (**a**) Datasets ($\dot{X}$ and X) are generated from the Lorenz 96 system. Based on X, Θ under $\Phi \in R^{1\times 66}$ is obtained. Then, feature selection based on sparse optimization is executed to select the contributing items in Φ, and a sparse basis matrix is acquired. Next, with the analysis of PCGC, the inferred network is compared with the benchmark network in terms of the PR and ROC curves. (**b**) Taking node no. 1 as an example, the regression equation underlying ${\dot{X}}_{1}$ and Θ is solved through sparse optimization. The nonzero elements in the vector of coefficient $a_{1}$ are fetched to form $\varphi_{1}$. (**c**) Then, the corresponding set of nodes $S_{1}$ is refined. Based on $S_{1}$, a nonlinear data matrix $\Theta_{1}^{\prime }$ is obtained from $X_{1}^{\prime }$ with the new basis function library $\Phi_{1}^{\prime }\in R^{1\times 15}$. The new regression equation underlying ${\dot{X}}_{1}$ and $\Theta_{1}^{\prime }$ is constructed. As a result, the final subnetwork of node no. 1 is judged using the PCGC analysis.

In general, the classical least squares method can be used to deal with Equations (5) and (6). However, the solution procedure for these equations may be problematic and inexact when the number of nonlinear basis functions is larger than the number of available observations. In fact, typically, not all basis function terms can influence the target variable. Most equations of real systems are governed by only a few relevant terms (or variables) that define the dynamical behaviors of target variables, which makes the system equations sparse in a high-dimensional space of a nonlinear basis function, especially for large networked systems. Actually, the consideration of all of the basis functions (including other unrelated terms) may lead to the deterioration of prediction performance in the whole system. In this case, Sparse Polynomial Conditional Granger Causality (SPCGC) is proposed, a flowchart of which is shown in Figure 2 based on the results of the simulation of a Lorenz 96 system with N=10 nodes. A benchmark network is obtained from the system model. Both the area under the receiver operating characteristic curve (AUROC) and the area under the precision–recall curve (AUPR) are utilized as standard metrics for evaluation.

The process of SPCGC can be divided into two steps. Datasets ($\dot{X}$ and X) are generated from the system model. Based on the datasets of X and the polynomial order *r*, a nonlinear data matrix Θ is obtained with the candidate basis function library Φ. For each target variable *i*, the regression model underlying the derivative ${\dot{X}}_{i}$ and nonlinear data matrix Θ is first formulated, and the coefficient $a_{i}$ of this can be solved using the sparse optimization shown in Equation (10).

(10) $${\hat{a}}_{i}=\underset{a_{i}}{\arg\min}\left({∥{\dot{X}}_{i}-\Theta a_{i}∥}_{2}^{2}+\lambda {∥a_{i}∥}_{1}\right),$$

where λ is a sparse penalty parameter. Based on the results of Equation (10), the basis function terms corresponding to the nonzero elements in the vector of coefficient $a_{i}$ are fetched to form a new set of basis functions. Then, we obtain the candidate variable sets for each ${\dot{X}}_{i}$ according to the new set of basis functions. Next, we rearrange Θ with the candidate variable sets, which are expressed as the new nonlinear data matrix $\Theta_{i}^{\prime }$, and reform the regression model of the PCGC model. Finally, we execute Granger causality analysis with the F-test in terms of the given significance level and confirm the causal variables of the target variable *i*. Another subnetwork can be inferred in a similar way. As a result, the whole network can be identified. The detailed procedure of the SPCGC algorithm can be illustrated as follows.

**Step 1:** Based on Equation (2), a feature selection method is first used to select the columns of Θ for dimensionality reduction. Actually, this is equivalent to selecting the candidate set of polynomial items from Φ. Here, the feature selection method is formulated based on the Lasso optimization in Equation (10). The candidate set of polynomial items *C* corresponds to the nonzero elements in the vector of coefficient $a_{i}$.

**Step 2:** Based on the candidate set of polynomial items *C*, the corresponding driving variables are denoted as $D_{j}$, j=1,2,…,K, where *K* is the number of driving variables fetched from *C*. The polynomial items related to $D_{j}$ in *C* are denoted as $C^{D_{j}}$.

**Step 3:** Based on the set of driving variables $D=\left\{D_{1},D_{2},\ldots ,D_{K}\right\}$, the corresponding unrestricted model can be reformulated. For each target variable $x_{i}$, let $y_{i}={\dot{X}}_{i}$; the corresponding unrestricted model is shown in Equation (11).

(11) $$y_{i}=\Theta_{D}a_{i}+\Xi_{i},$$

where $\Theta_{D}$ is the nonlinear data matrix with the polynomial basis of driving variables *D*. Then, the residual $\Xi_{i}$ can be calculated as follows:

(12) $$\Xi_{i}=y_{i}-\Theta_{D}\Theta_{D}^{†}y_{i},$$

where the pseudo-inverse $\Theta_{D}^{†}={\left(\Theta_{D}^{T}\Theta_{D}\right)}^{-1}\Theta_{D}^{T}$.

**Step 4:** Then, the restricted model should be formulated as shown in Equation (13).

(13) $$y_{i}=\Theta_{D\backslash D_{j}}{a^{\prime }}_{i}+{\Xi^{\prime }}_{i},$$

where $D\backslash D_{j}=\left\{D_{1},D_{2},\ldots ,D_{j-1},D_{j+1},\ldots ,D_{K}\right\}$, and $D\backslash D_{j}$ means removing all of the related polynomial items $C^{D_{j}}$ in the candidate set *C*. Then, $\Theta_{D\backslash D_{j}}$ is constructed based on the basis $C^{D\backslash D_{j}}$. So, for each $D_{j}$, the residual ${\Xi^{\prime }}_{i}$ can be calculated as follows:

(14) $${\Xi^{\prime }}_{i}=y_{i}-\Theta_{D\backslash D_{j}}\Theta_{D\backslash D_{j}}^{†}y_{i},$$

where the pseudo-inverse $\Theta_{D\backslash D_{j}}^{†}={\left(\Theta_{D\backslash D_{j}}^{T}\Theta_{D\backslash D_{j}}\right)}^{-1}\Theta_{D\backslash D_{j}}^{T}$.

**Step 5:** In order to judge the causal relationship between node $D_{j}$ and node *i*, the PCGC index is calculated as follows:

(15) $$PCGC_{D_{j}\to x_{i}}=\ln\frac{\sigma^{2}\left({\Xi^{\prime }}_{i}\right)}{\sigma^{2}\left(\Xi_{i}\right)}.$$

If $\sigma^{2}\left({\Xi^{\prime }}_{i}\right)>\sigma^{2}\left(\Xi_{i}\right)$, i.e., removing the effect of $D_{j}$ will increase the error variance of the prediction of $x_{i}$, there exists a link $D_{j}\to x_{i}$. A positive threshold 0<δ<1 can be set for the extraction of causal links with larger Granger causal weights. In addition, F-statistic tests can be performed to confirm the significance level for each link.

### 2.3. Selection of the Polynomial Order *r* and Sparse Penalty Parameter λ

As is well known, for the determination of models that provide better predictions, overfitting and generalization are critical factors for judging which model performs better. Cross-validation and regularization are two classical ways of restraining the complexity of a model. In our work, the performance of our proposed methods (PCGC and SPCGC) is also based on the selection of the following parameters: the polynomial order *r* and the sparse penalty parameter λ, respectively. Although these parameters can be selected according to practical experience, an automatic selection scheme should be introduced and analyzed.

For PCGC, in order to ensure the fitting precision of nonlinear system equations and avoid overfitting, a proper polynomial order *r* is vital for dynamical prediction and Granger causality analysis. Here, *K*-fold cross-validation is adopted. Firstly, a cross-validation partition is created for data, and this can be used to define a specified size of training sets and test sets for validating the effectiveness of a statistical model based on cross-validation. In detail, all *M* observations are divided into *K* disjoint subsamples (folds) using a random partition with a roughly equal size. Then, through randomly repeated cross-validations, we minimize the cost function of the error to determine the best model.

In addition, for the simulation of SPCGC, the selection of the sparse penalty parameter λ in the regularization is important. Here, the Hannan–Quinn criterion (HQC) [49] can be used for the selection of the sparse penalty parameter. In detail, the HQC is adopted to balance the model complexity and model accuracy and to seek the best model’s ability to describe datasets. For our case, based on the basic idea of the HQC, its detailed definition for the selection of λ is shown as follows:

(16) $$HQC\left(\lambda \right)=\ln\left|\sigma_{\lambda }^{2}\right|+\frac{2n_{\lambda }\ln\left(\ln M\right)}{M},$$

where *M* is the size of the data, $n_{\lambda }$ is the number of variables, and $\sigma_{\lambda }^{2}$ is the variance in the error. $n_{\lambda }$ and $\sigma_{\lambda }^{2}$ can be computed for each value of λ to search for the optimal solution.

## 3. Results

In order to verify the performance of our proposed methods, different nonlinear system models were collected and employed, including dynamical trajectories displaying transients towards steady states, dynamical trajectories exhibiting transients toward periodic dynamics, non-periodic dynamical trajectories, and chaotic dynamical trajectories [17].

These different nonlinear systems were categorized and verified according to their different types of dynamical trajectories. For dynamical trajectories from transients towards steady states, we employed a gene regulation network model with Michaelis Menten kinetics [50] and a metabolic network model (yeast glycolysis) [15]. For dynamical trajectories from transients to periodic dynamics, we simulated another two biological models: another metabolic network model (glycolytic oscillator) [13,17,51,52,53] and a biochemical reaction network model [14]. For non-periodic dynamical trajectories, we adopted two networked models: a Kuramoto oscillator and a phase-coupled oscillator [17]. For chaotic dynamical trajectories, we tested the Lorenz oscillator system and Rössler oscillator system. To verify the reconstruction performance of the larger networks, we also utilized the Lorenz 96 networked system. All of the system models are described in Appendix A.

### 3.1. Detailed Case Description of PCGC: Glycolytic Oscillator Network

In this section, we consider the inference of a metabolic network based on the glycolytic oscillator model. The complex formulations of the equations in this model are composed of constant, polynomial, and fractional terms. This is a classical benchmark problem for model prediction, automatic inference, and system identification. The glycolytic oscillator model is set to describe the dynamical behavior of metabolic networks. Accelerating the inference of metabolic networks would aid interventions in metabolic diseases.

**Figure 3:**
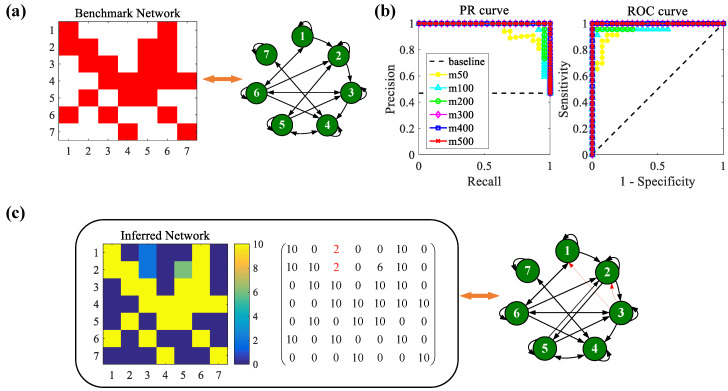
Simulation results for the glycolytic oscillator model. Here, T=2, r=5, $P_{val}=10^{-10}$, and σ=0.01. (**a**) Benchmark network together with the corresponding directed graph. (**b**) The curves of the ROC and PR with different numbers of samples. (**c**) Discovery rate matrix inferred from 10 realizations and the corresponding directed graph.

First, to illustrate the effectiveness of our proposed PCGC method in the case of dynamical trajectories from transients to periodic dynamics, the following simulation is based on the nonlinear dynamical equations of a glycolytic oscillator with n=7 nodes (variables). In Figure 3a, a benchmark adjacency matrix and a directed graph are drawn from the given equations of the system model. For the adjacency matrix, the red squares are existent links among nodes, and the white areas are nonexistent links.

Then, synthetic datasets are derived, the related parameters of which in the simulation are shown in Figure 3. In Figure 3b, the curves of the ROC and PR are plotted with different numbers of observations. Here, the number of time points T=2 is kept unchanged, and the number of measurements is taken from the set m={50,100,200,300,400,500}. We can see that the values of the AUROC and AUPR are relatively lower when m=50. As *m* increases, the performance becomes better and better, and perfect reconstruction is realized when m={300,400,500}. Next, the performance of robust estimation with noise is investigated. The Gaussian noise variance is set to σ=0.01. In this case, multiple realizations are carried out, and the number of realizations is set to 10. Based on the PCGC analysis with $P_{val}=10^{-10}$, the final network is inferred. In Figure 3c, the discovery rate matrix inferred from 10 realizations in the glycolytic oscillator model is demonstrated (the discovery rate means the total number of links that are rightly discovered over multiple realizations). It is obvious that the true positive links are always found, except for link 5→2, which has a 60% discovery rate. Meanwhile, there are two false positive links with a 20% discovery rate (3→1 and 3→2). In Figure 3c the final directed graph is plotted from the discovery rate matrix, where the thickness of the line indicates the value of the discovery rate. In general, the results as a whole demonstrate the robust performance of the PCGC method.

### 3.2. Comparison of PCGC and SPCGC: Gene Regulatory Network

As mentioned above, PCGC is a method based on a nonlinear regression model. If the number of nonlinear basis functions is greater than the number of observations, the regression equation for Granger causality analysis is an underdetermined problem, and the exact solution will not be obtained. In order to verify the validity and necessity of SPCGC, a comparison between PCGC and SPCGC is made by executing a simulation of a gene regulatory dynamical system with Michaelis Menten kinetics. We first apply our proposed methods to reconstruct a gene regulatory network with N=10 nodes. The generation of a benchmark network follows the mechanism using an approximate power-law output degree distribution with an average degree of $\langle k\rangle =3$. Then, synthetic datasets with M=mT observations are obtained. Here, the time points are kept unchanged at each measurement (T=2). In order to investigate how the number of observations influences the performance of our method, the number of multiple measurements *m* is set to 400 and 700. Other related parameters are given as follows: Gaussian noise strength σ=0.01, the polynomial order r=5, and the sparse penalty parameter λ=0.001.

**Figure 4:**
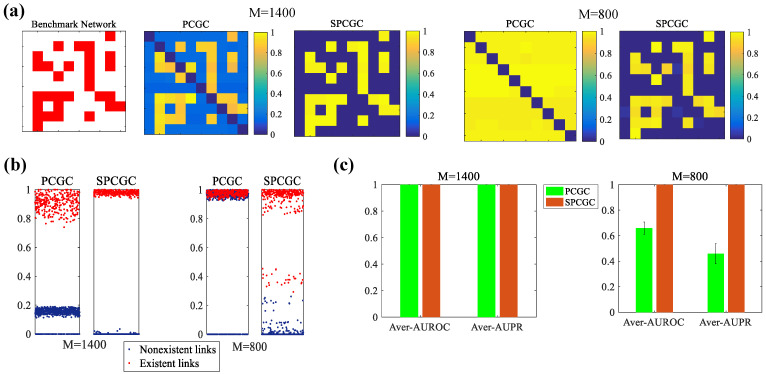
Simulation results for the comparison of PCGC and SPCGC in different cases by using M=1400 and M=800 observations. (**a**) The benchmark network of a gene regulation network with N=10 nodes and the Granger causality index matrixes obtained by using M=1400 and M=800 observations. (**b**) Granger causality indexes of reconstructed elements in the matrixes recovered using PCGC and SPCGC over 10 realizations. (**c**) The average values with standard deviations of the AUROC and AUPR over 10 realizations.

In Figure 4a, the Granger causality matrixes of PCGC and SPCGC are demonstrated in the cases of M=1400 and M=800 observations, respectively. With adequate observations (M=1400), the two methods could find all existent links, especially the existent links in the Granger causality matrix of SPCGC with a higher causal strength. For PCGC, compared with SPCGC, the causal strength of many existent links is relatively smaller. Meanwhile, all of the nonexistent links have a small causal strength, which can be eliminated by setting a specific threshold. In this case, both PCGC and SPCGC can achieve a perfect reconstruction. With insufficient observations (M=800), PCGC obviously loses its function in reconstructing the network, as all of the links in the Granger causality matrix of PCGC have a high causal strength. However, although there are a few nonexistent links with slight causal intensity, SPCGC still maintains a good performance. In the following section, the performance of robust estimation with different numbers of samples is explored. Robust estimation is verified by running over 10 realizations. In particular, the elements in the Granger causality matrixes reconstructed using PCGC and SPCGC methods over 10 realizations are exhibited in Figure 4b. As shown in Figure 4b, both PCGC and SPCGC achieve perfect reconstruction when M=1400. The existent links and nonexistent links can be divided using a given threshold. When M=800, we find that PCGC largely mixes existent links with nonexistent links. This is short of a certain threshold for distinguishing existent links from nonexistent links. However, in this case, an almost perfect separation by SPCGC can also be seen when the number of samples is smaller than the number of nonlinear basis functions. The average values (standard deviations) of the AUROC and AUPR over 10 realizations are computed and shown in Figure 4c. Overall, it is worth emphasizing that SPCGC demonstrates perfect performance with different numbers of observations, even when the number of samples is smaller than the number of nonlinear basis functions, i.e., the regression equation is underdetermined.

### 3.3. Robust Results of Classical Models in Different Cases

To further assess the performance of our proposed method, both the AUROC and the AUPR were utilized to evaluate our proposed method in different classical models. These models include a biochemical reaction network, glycolytic oscillator network, yeast glycolysis network, gene regulatory network, Kuramoto oscillator network, and phase-coupled oscillator network, as well as the Lorenz 96, Lorenz, and Rössler networks.

**Figure 5:**
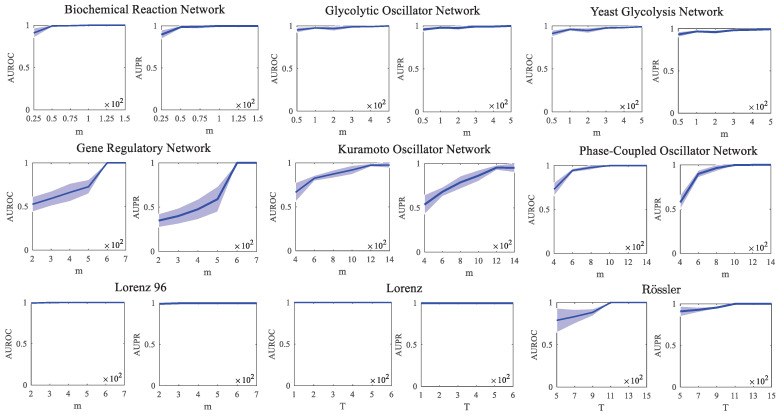
Simulation results of different models with different numbers of observations (*m* or *T*). All of the values of the AUROC and AUPR are averaged over 10 realizations. The blue-shaded area is the error bound of the AUROC or AUPR over 10 realizations.

Firstly, the simulation of different models with different numbers of observations was carried out. The detailed parameter settings of the simulations of these different models for different numbers of observations are shown in Table 1, and they include *m*, *T*, σ, and *r*. The values of the average AUROC and average AUPR were computed by averaging over 10 independent realizations. In detail, the inference results of different models under different conditions were explored. The different conditions involve only one parameter being changed while the others are kept the same. In Table 1, the “-” sign means that these parameters vary in the simulations. Here, a different number of observations (*m* or *T*) is used for detailed comparisons, and the specific ranges of values are shown in Figure 5. As is known, most of the benchmark network topologies can be derived from system models, except for the gene regulatory network, Kuramoto oscillator network, and phase-coupled oscillator network. The network topologies of these three models should be given in advance as the benchmark networks. Here, the preset topology follows the scale-free generative mechanism, in which the output degree of the network approximates a popular power-law degree distribution with N=10 and $\langle k\rangle =3$. For the PCGC method, the simulation results of different models with different numbers of observations are shown in Figure 5. As shown in Figure 5, the performance trend rises overall with the increase in the number of samples. As we have seen, with a certain number of samples, nearly perfect reconstruction is realized.

**Figure 6:**
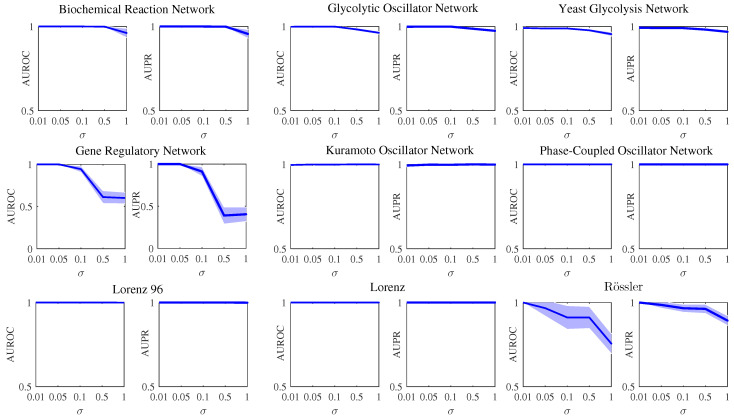
Simulation results of different models at different noise intensities σ. All of the values of the AUROC and AUPR are averaged over 10 realizations. The blue-shaded area is the error bound of the AUROC or AUPR over 10 realizations.

Next, in order to further address the robustness to uncertainties in data, a simulation with different noise intensities σ should be considered for the further verification of PCGC. The simulation results of different models at different noise intensities σ are shown in Figure 6. All of the values of the AUROC and AUPR are averaged over 10 realizations. The related parametric settings of the simulations in Figure 6 are listed in Table 2. The values of *m*, *T*, and *r* are fixed, while σ is taken from the set {0.01;0.05;0.1;0.5;1}. With the increase in σ, the performance of PCGC on these classical system models is generally stable, except for the gene regulation network and Rössler oscillator system. In the simulation of the gene regulation network and Rössler oscillator, the performance of PCGC goes down quite sharply with the increase in σ, especially for the gene regulation network. For the Kuramoto oscillator, phase-coupled oscillator, Lorenz oscillator, and Lorenz 96 model, both the average AUROC and average AUPR of PCGC over 10 realizations basically stay at the maximum value, which indicates that PCGC has strong robustness in the case of different values of σ. For the biochemical reaction network, glycolytic oscillator network, and yeast glycolysis network, the performance of PCGC slightly decreases when σ is high. In general, the robustness of PCGC is demonstrated in the case of different values of σ.

**Figure 7:**
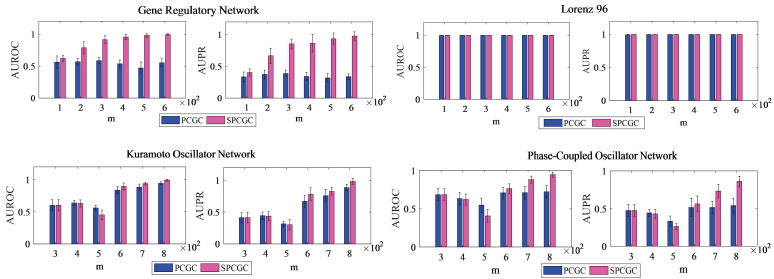
Simulation results of different models for the comparison of PCGC and SPCGC. All of the values of the AUROC and AUPR are averaged over 10 realizations.

Finally, in the case of a higher polynomial order with relatively insufficient samples, the nonlinear regression equation for PCGC will be underdetermined. Then, high performance will be not guaranteed. To some extent, it will even become very poor. Meanwhile, even if the number of variables (nodes) is small, the polynomial space might also be very large with a higher polynomial order. In this way, it is necessary to propose SPCGC for dimension reduction to improve the reconstruction performance. Simulations comparing the PCGC and SPCGC methods are demonstrated in Figure 7. Here, Lorenz 96, the gene regulatory network, the Kuramoto oscillator network, and the phase-coupled oscillator network are taken for further investigation; the network generation mechanism and network parameter settings are the same as those in the simulation in Figure 5. The other parameters for the simulations in Figure 7 are listed in Table 3. As shown in Figure 7, based on the average AUROC and average AUPR over multiple realizations, the performance of SPCGC is higher than that of PCGC as a whole. In particular, for the Lorenz 96 network, unlike PCGC, SPCGC has almost the same values for the AUROC and AUPR. However, the final reconstructed network of SPCGC has more exact and reliable causal weights with higher confidence levels. In this case, a similar comparison between SPCGC and PCGC can be performed by referring to the analysis in Figure 4.

## 4. Discussion

The traditional Granger causality method was proposed based on a linear VAR model, which often cannot achieve good results under the condition of an insufficient sample size. Based on the consideration of nonlinearity, sparsity, and causality, the model-free PCGC and SPCGC methods are presented in this study and, based on the simulation of different complex nonlinear networked systems, the robustness and effectiveness of our proposed method are verified. However, the following questions still need to be addressed and discussed.

(1) The heterogeneity and diversity of data can guarantee the performance and quality of causal inference methods. Therefore, different initial values under different conditions are used to collect data using a scheme with multiple measurements. In this case, our proposed methods are generally proven to be effective and robust in simulations of many system models. However, some issues should be addressed. In Figure 5, for the gene regulatory network and Kuramoto oscillator network, when the sample size is relatively small, the heterogeneity of the data is not adequate. This is also the reason why our proposed method PCGC obtains lower AUROC and AUPR values in the simulations of the gene regulatory network and Kuramoto oscillator network. However, as the sample size increases, the performance of PCGC is greatly improved. On the other hand, the Lorenz 96 and the Lorenz systems have chaotic dynamic trajectories. The data generated from them are heterogeneous enough. So, PCGC still achieves good performance even with a relatively smaller sample size in the simulation of Lorenz 96 and the Lorenz systems. The AUROC and AUPR of PCGC reach stable values as the sample size increases. In particular, we conducted a detailed comparison between PCGC and SPCGC in a gene regulatory network, as shown in Figure 4. The performance of methods varies greatly as the sample size increases. We attempted to analyze the reasons in detail to make this study more representative. Of course, other models can be used for the same analysis and comparison.

(2) The focus of our theoretical research is not to specifically study the impact of the presence of measurement noise on Granger causality inference, but to consider the robustness of our method in the presence of certain measurement noise. For this reason, we also performed some simulation studies in the case of different noise intensities. The robustness to uncertainties in the data was addressed; see Figure 6. Figure 6 provides the simulation results of different models at different noise intensities. We discuss this problem of measurement noise on Granger causality analysis [54,55] and refer to some works that have already attempted to explore this aspect [56,57]. Based on the consideration of noise, we believe that a combination of state-space modeling and some filtering methods for our PCGC framework would work well.

(3) To improve the performance of our proposed method, the choice of method parameters is also a crucial issue. In general, for a complex system, the same polynomial order *r* can be set for all system equations. In particular, for most networked systems, due to the difference in the complexity of the system equation for each node, it is necessary to give a specific polynomial order to different system equations. In this way, the respective complexity of the system equations can be roughly estimated by determining different polynomial orders of the system equations. Similarly, for a complex system, the same penalty parameter λ can be set for all system equations. For different equations of a system with heterogeneous complexity, it is more reasonable to choose different values of λ for different target nodes (with the respective equations of the system model) rather than using the same value of λ.

(4) In our study, we specifically focus on the proposed methods to verify their performance from different perspectives in detail, such as different types of models, different types of data, different sample sizes, and different noise intensities. In the future, the proposed methods will be compared and analyzed with existing advanced causal network inference methods. For further perspectives, the Lasso selection in SPCGC can be replaced with other compressed sensing algorithms for the investigation of better performance. We can make a similar comparison of penalized regression methods, as shown in [44]. On the other hand, based on a summary of some existing Granger causality methods [38,39,40], including an analysis of causal methods based on information theory [58,59], we should obtain an idea of solving causal network inference. In particular, a similar method should be pointed out, as it was proposed based on software space intervention analysis, and it is named algorithmic information dynamics. It might be considered as an alternative to our causal approach, but it uses computer programs rather than polynomial differential equations [59], and this needs to be studied in our future work.

## 5. Conclusions

In brief, the exact identification and dynamic prediction of causal networks from data were demonstrated by our proposed methods, one of which is termed Polynomial Conditional Granger Causality (PCGC), while the other is its expanded sparse-promoting version (SPCGC). These methods do not require assumptions on the specific form of the nonlinear system equations. In particular, a general polynomial approximation is adopted to fit the relationships among nodes, which has the superiority of turning the nonlinearity of complex systems into some additive terms. Then, SPCGC combines nonlinear regression based on Granger causality with sparse optimization, which is fit for larger high-dimensional systems with a relatively smaller number of samples.

From the point of view of network science, all real systems can be represented by networks. Due to the complexity of real systems, the mathematical model of a real system is often indistinct or unavailable. Therefore, for networked systems, it is necessary to develop a data-driven causal inference method that can provide a foundation for subsequent network analysis. In general, as a simple and effective method, this model-free framework can, to some extent, provide new prospects, and represents an attempt to develop a basis for network reconstruction methods in the future.

## Figures and Tables

**Table 1:** Parametric settings of simulations of different models with different numbers of observations. Among these parameters, *m* is the number of measurements, *T* is the number of time points at each measurement, σ is the strength of Gaussian noise, and *r* is the polynomial order. Based on *m* and *T*, the total number of observations *M* is collected (M=mT). The “-” sign means that parameters vary in the simulations.

Model	*m*	*T*	σ	*r*
Lorenz oscillator	1	-	0.01	5
Rössler oscillator	1	-	0.01	5
Lorenz 96	-	2	0.01	4
Biochemical reaction network	-	2	0.01	4
Glycolytic oscillator	-	2	0.01	5
Yeast glycolysis	-	2	0.01	5
Gene regulatory network	-	2	0.01	4
Kuramoto oscillator	-	2	0.01	4
Phase-coupled oscillator	-	2	0.01	4

**Table 2:** Parametric settings of simulations of different models with different noise intensities σ.

Model	*m*	*T*	σ	*r*
Lorenz oscillator	1	600	{0.01;0.05;0.1;0.5;1}	5
Rössler oscillator	1	1500	{0.01;0.05;0.1;0.5;1}	5
Lorenz 96	700	2	{0.01;0.05;0.1;0.5;1}	4
Biochemical reaction network	150	2	{0.01;0.05;0.1;0.5;1}	4
Glycolytic oscillator	500	2	{0.01;0.05;0.1;0.5;1}	5
Yeast glycolysis	500	2	{0.01;0.05;0.1;0.5;1}	5
Gene regulatory network	700	2	{0.01;0.05;0.1;0.5;1}	4
Kuramoto oscillator	1400	2	{0.01;0.05;0.1;0.5;1}	4
Phase-coupled oscillator	1400	2	{0.01;0.05;0.1;0.5;1}	4

**Table 3:** Parametric settings of simulations of different models for the comparison of PCGC and SPCGC. Here, *m* is the number of measurements, *T* is the number of time points at each measurement, σ is the strength of noise, *r* is the polynomial order, and λ is the sparse penalty parameter.

Model	*m*	*T*	σ	*r*	λ
Gene regulatory network	{100; 200; 300; 400; 500; 600}	2	0.01	5	0.002
Lorenz 96	{100; 200; 300; 400; 500; 600}	2	0.01	5	0.001
Kuramoto oscillator	{300; 400; 500; 600; 700; 800}	2	0.01	5	0.003
Phase-coupled oscillator	{300; 400; 500; 600; 700; 800}	2	0.01	5	0.003

## Data Availability

The original contributions presented in the study are included in this article, further information can be available on request from the corresponding author.

## Appendix A. Nonlinear System Models for Simulation

### Appendix A.1. Lorenz Oscillator

The system model of the Lorenz oscillator is formulated as shown in Equation (A1).

(A1) $$\begin{array}{c} \dot{x}=\sigma \left(y-x\right)+\xi_{x}\;\\ \dot{y}=x\left(\rho -z\right)-y+\xi_{y}\;\\ \dot{z}=xy-\beta z+\xi_{z} \end{array}$$

where the standard parameters are σ=10, ρ=28, and β=8/3. $\xi_{x}$, $\xi_{y}$, and $\xi_{z}$ are zero-mean uncorrelated Gaussian noise terms with identical unit variance ($\xi ∼N(0,\sigma^{2})$).

### Appendix A.2. Rössler Oscillator

The system model of the Rössler oscillator is formulated as shown in Equation (A2).

(A2) $$\begin{array}{c} \dot{x}=-y-z+\xi_{x}\;\\ \dot{y}=x+ay+\xi_{y}\;\\ \dot{z}=b+z\left(x-c\right)+\xi_{z} \end{array}$$

where a=0.1, b=0.1, and c=14. $\xi_{x}$, $\xi_{y}$, and $\xi_{z}$ are zero-mean uncorrelated Gaussian noise terms with identical unit variance ($\xi ∼N(0,\sigma^{2})$).

### Appendix A.3. Lorenz 96

The Lorenz 96 model is a dynamical system formulated by Edward Lorenz in 1996, and it is defined as shown in Equation (A3).

(A3) $${\dot{x}}_{i}=\left(x_{i+1}-x_{i-2}\right)x_{i-1}-x_{i}+F+\xi_{i},$$

where i=1,2,…,N; $x_{-1}=x_{N-1}$, $x_{0}=x_{N}$, and $x_{1}=x_{N+1}$. $x_{i}$ is the state of the system, and *F* is a forcing constant. F=8 is a common value known to cause chaotic behavior. $\xi_{i}$ is a zero-mean uncorrelated Gaussian noise term with identical unit variance ($\xi_{i}∼N\left(0,\sigma^{2}\right)$).

### Appendix A.4. Biochemical Reaction Network

The system model of the biochemical reaction network is formulated as shown in Equation (A4).

(A4) $$\begin{array}{c} {\dot{x}}_{1}=-\gamma_{1}x_{1}+\frac{\alpha_{1}}{1+x_{6}^{n_{1}}}+\xi_{1}\;\\ {\dot{x}}_{2}=-\gamma_{2}x_{2}+\frac{\alpha_{2}}{1+x_{4}^{n_{2}}}+\xi_{2}\;\\ {\dot{x}}_{3}=-\gamma_{3}x_{3}+\frac{\alpha_{3}}{1+x_{5}^{n_{3}}}+\xi_{3}\;\\ {\dot{x}}_{4}=-\gamma_{4}x_{4}+\beta_{1}x_{1}+\xi_{4}\;\\ {\dot{x}}_{5}=-\gamma_{5}x_{5}+\beta_{2}x_{2}+\xi_{5}\;\\ {\dot{x}}_{6}=-\gamma_{6}x_{6}+\beta_{3}x_{3}+\xi_{6} \end{array}$$

where $x_{1}$, $x_{2}$, and $x_{3}$ are concentrations of the mRNA transcripts of genes 1, 2, and 3, respectively; $x_{4}$, $x_{5}$, and $x_{6}$ are concentrations of the proteins of genes 1, 2, and 3, respectively; $\alpha_{1}$, $\alpha_{2}$, and $\alpha_{3}$ are the maximum promoter strengths for the corresponding genes; $\gamma_{1}$, $\gamma_{2}$, and $\gamma_{3}$ are mRNA decay rates; $\gamma_{4}$, $\gamma_{5}$, and $\gamma_{6}$ are protein decay rates; $\beta_{1}$, $\beta_{2}$, and $\beta_{3}$ are protein production rates; and $n_{1}$, $n_{2}$, and $n_{3}$ are hill coefficients. $\xi_{i}$ is a zero-mean uncorrelated Gaussian noise term with identical unit variance ($\xi_{i}∼N\left(0,\sigma^{2}\right)$). The parameters of Equation (A4) are given in Table A1.

**Table A1:** Parameter settings in the biochemical reaction network.

$\alpha_{1}=4$	$\alpha_{2}=3$	$\alpha_{3}=5$			
$\gamma_{1}=0.3$	$\gamma_{2}=0.4$	$\gamma_{3}=0.5$	$\gamma_{4}=0.2$	$\gamma_{5}=0.4$	$\gamma_{6}=0.6$
$\beta_{1}=1.4$	$\beta_{2}=1.5$	$\beta_{3}=1.6$			
$n_{1}=4$	$n_{2}=4$	$n_{3}=4$			

### Appendix A.5. Metabolic Network 1: Glycolytic Oscillator

The system model of the glycolytic oscillator is formulated as shown in Equation (A5).

(A5) x˙1=J0−k1x1x61+x6x6K1K14+ξ1x˙2=2k1x1x61+x6x6K1K14−k2x2N−x5−k6x2x5+ξ2x˙3=k2x2N−x5−k3x3A−x6+ξ3x˙4=k3x3A−x6−k4x4x5−κx4−x7+ξ4x˙5=k2x2N−x5−k4x4x5−k6x2x5+ξ5x˙6=−2k1x1x61+x6x6K1K14+2k3x3A−x6−k5x6+ξ6x˙7=ψκx4−x7−kx7+ξ7

where $x_{1}$ is the concentration of glucose; $x_{2}$ is a pool compounded of triose phosphates, glyceraldehyde 3-phosphate, and dihydroxyacetone phosphate; $x_{3}$ is the concentration of 1,3-bisphosphoglycerate; $x_{4}$ is a pooled compound of pyruvate and acetaldehyde; $x_{5}$ is the concentration of NADH; $x_{6}$ is the concentration of ATP; and $x_{7}$ is the concentration of extracellular coupling substances [51,52]. $\xi_{i}$ is a zero-mean uncorrelated Gaussian noise term with identical unit variance ($\xi_{i}∼N\left(0,\sigma^{2}\right)$). The parameters of Equation (A5) are given in Table A2.

**Table A2:** Parameter settings in the glycolytic oscillator model.

$J_{0}=2.5$;	k=1.8;	ψ=0.1;	κ=13	
$K_{1}=0.52;$	N=1;	A=4;	$k_{1}=100$	
$k_{2}=6$;	$k_{3}=16$;	$k_{4}=100$;	$k_{5}=1.28$;	$k_{6}=12$

### Appendix A.6. Metabolic Network 2: Yeast Glycolysis

The system model of yeast glycolysis is formulated as shown in Equation (A6).

(A6) $$\begin{array}{c} {\dot{x}}_{1}=a_{1}+\frac{a_{2}x_{1}x_{6}}{1+a_{3}x_{6}^{4}}+\xi_{1}\;\\ {\dot{x}}_{2}=\frac{b_{1}x_{1}x_{6}}{1+b_{2}x_{6}^{4}}+b_{3}x_{2}-b_{4}x_{2}x_{7}+\xi_{2}\;\\ {\dot{x}}_{3}=c_{1}x_{2}+c_{2}x_{3}+c_{3}x_{2}x_{7}+c_{4}x_{3}x_{6}+\xi_{3}\;\\ {\dot{x}}_{4}=d_{1}x_{3}+d_{2}x_{4}+d_{3}x_{5}+d_{4}x_{3}x_{6}+d_{5}x_{4}x_{7}+\xi_{4}\;\\ {\dot{x}}_{5}=e_{1}x_{4}+e_{2}x_{5}+\xi_{5}\;\\ {\dot{x}}_{6}=\frac{f_{1}x_{1}x_{6}}{1+f_{2}x_{6}^{4}}+f_{3}x_{3}+f_{4}x_{3}x_{7}+f_{5}x_{6}+\xi_{6}\;\\ {\dot{x}}_{7}=g_{1}x_{2}+g_{2}x_{2}x_{7}+g_{3}x_{4}x_{7}+\xi_{7} \end{array}$$

where the description of $x_{i}$ (i=1,2,…,7) is the same as above. $\xi_{i}$ is a zero-mean uncorrelated Gaussian noise term with identical unit variance ($\xi_{i}∼N\left(0,\sigma^{2}\right)$). The parameters of Equation (A6) are given in Table A3.

**Table A3:** Parameter settings in the yeast glycolysis model.

$a_{1}=2.5$;	$a_{2}=-100$;	$a_{3}=13.6769$		
$b_{1}=200$;	$b_{2}=13.6769$;	$b_{3}=-6$;	$b_{4}=-6$	
$c_{1}=6$;	$c_{2}=-64$;	$c_{3}=6$;	$c_{4}=16$	
$d_{1}=64$;	$d_{2}=-13$;	$d_{3}=13$;	$d_{4}=-16$;	$d_{5}=-100$
$e_{1}=1.3$;	$e_{2}=-3.1$			
$f_{1}=-200$;	$f_{2}=13.6769$;	$f_{3}=128$;	$f_{4}=-1.28$;	$f_{5}=-32$
$g_{1}=6$;	$g_{2}=-18$;	$g_{3}=-100$		

### Appendix A.7. Gene Regulatory Network

Suppose that gene interactions are modeled by the Michaelis–Menten equation, as shown in Equation (A7). This is a popular system of equations for gene networks.

(A7) $${\dot{x}}_{i}=-x_{i}^{f}+\sum_{j=1}^{N}b_{ij}\frac{x_{j}^{h}}{x_{j}^{h}+1}+\xi_{i},$$

where f=1 and h=2. Let $B={\left[b_{ij}\right]}_{N\times N}$ be the adjacency matrix, which characterizes the interactions among nodes. $b_{ij}\neq 0$ if the *j*-th variable connects to the *i*-th one; otherwise, $b_{ij}=0$. $\xi_{i}$ is a zero-mean uncorrelated Gaussian noise term with identical unit variance ($\xi_{i}∼N\left(0,\sigma^{2}\right)$).

### Appendix A.8. Kuramoto Oscillator

The system model of the Kuramoto oscillator is formulated as shown in Equation (A8).

(A8) $$\begin{array}{c} {\dot{x}}_{i}=\omega_{i}+\sum_{j=1}^{N}b_{ij}\sin\left(x_{j}-x_{i}\right)+\xi_{i}, \end{array}$$

where $\omega_{i}$ is a constant natural frequency. The second term describes the interactions between variable *i* and its interacting partners *j*. The nonlinear functions $sin\left(•\right)$ represent the dynamical laws that govern the variables of the system. $B={\left[b_{ij}\right]}_{N\times N}$ is an adjacency matrix that describes the interactions among variables. $b_{ij}\neq 0$ if the *j*-th variable connects to the *i*-th one; otherwise, $b_{ij}=0$. $\xi_{i}$ is a zero-mean uncorrelated Gaussian noise term with identical unit variance ($\xi_{i}∼N\left(0,\sigma^{2}\right)$).

### Appendix A.9. Phase-Coupled Oscillator

The model of a phase-coupled oscillator is given with coupling functions that have two Fourier items in Equation (A9).

(A9) $$\begin{array}{c} {\dot{x}}_{i}=\omega_{i}+\sum_{j=1}^{N}b_{ij}\left[\sin\left(x_{j}-x_{i}-1.05\right)+0.33\sin\left(2\left(x_{j}-x_{i}\right)\right)\right]+\xi_{i}, \end{array}$$

where $\omega_{i}$ is a constant natural frequency. The two Fourier modes describe the interactions between variable *i* and its interacting partners *j*. The nonlinear functions $sin\left(•\right)$ represent the dynamical laws that govern the variables of the system. $B={\left[b_{ij}\right]}_{N\times N}$ is an adjacency matrix that describes the interactions among variables. $b_{ij}\neq 0$ if the *j*-th variable connects to the *i*-th one; otherwise, $b_{ij}=0$. $\xi_{i}$ is a zero-mean uncorrelated Gaussian noise term with identical unit variance ($\xi_{i}∼N\left(0,\sigma^{2}\right)$).
